# Reconstruction of GRACE Mass Change Time Series Using a Bayesian Framework

**DOI:** 10.1029/2021EA002162

**Published:** 2022-07-07

**Authors:** Ashraf Rateb, Alexander Sun, Bridget R. Scanlon, Himanshu Save, Emad Hasan

**Affiliations:** ^1^ Bureau of Economic Geology University of Texas at Austin Austin TX USA; ^2^ Center for Space Research University of Texas at Austin Austin TX USA

**Keywords:** Geodesy, GRACE (‐FO), Bayesian inference, MCMC, Mass change, Hydrology

## Abstract

Gravity Recovery and Climate Experiment and its Follow On (GRACE (‐FO)) missions have resulted in a paradigm shift in understanding the temporal changes in the Earth's gravity field and its drivers. To provide continuous observations to the user community, missing monthly solutions within and between GRACE (‐FO) missions (33 solutions) need to be imputed. Here, we modeled GRACE (‐FO) data (196 solutions) between 04/2002–04/2021 to infer missing solutions and derive uncertainties in the existing and missing observations using Bayesian inference. First, we parametrized the GRACE (‐FO) time series using an additive generative model comprising long‐term variability (secular trend + interannual to decadal variations), annual, and semi‐annual cycles. Informative priors for each component were used and Markov Chain Monte Carlo (MCMC) was applied to generate 2,000 samples for each component to quantify the posterior distributions. Second, we reconstructed the new data (229 solutions) by joining medians of posterior distributions of all components and adding back the residuals to secure the variability of the original data. Results show that the reconstructed solutions explain 99% of the variability of the original data at the basin scale and 78% at the one‐degree grid scale. The results outperform other reconstructed data in terms of accuracy relative to land surface modeling. Our data‐driven approach relies only on GRACE (‐FO) observations and provides a total uncertainty over GRACE (‐FO) data from the data‐generation process perspective. Moreover, the predictive posterior distribution can be potentially used for “nowcasting” in GRACE (‐FO) near‐real‐time applications (e.g., data assimilations), which minimize the current mission data latency (40–60 days).

## Key Points

- Uncertainties in existing and missing solutions of Gravity Recovery and Climate Experiment and its Follow On (GRACE (‐FO)) missions need to be informed
- Bayesian inference is used to decompose and model temporal GRACE (‐FO) signals
- The new data explain the variability in original observations and can be used for nowcasting

## 1 Introduction

Since 2002, the Gravity Recovery And Climate Experiment (GRACE) and its Follow‐On (FO) missions have resulted in a paradigm shift in our understanding of the changes in the Earth's gravity field and associated regional and global mass change and transport (Abich et al., 2019; Landerer et al., 2020; Tapley et al., 2004). GRACE (‐FO) missions provide monthly observations of mass changes caused by land water storage (Rodell et al., 2018; Scanlon et al., 2019), ocean currents (Adhikari et al., 2019; Chambers, 2006), ice sheets, and glaciers (Velicogna et al., 2020) which enable us to constrain the dynamics of these systems and assess impacts of climate change and human interventions (Reager et al., 2016; Rodell et al., 2018; Scanlon et al., 2021; Scanlon et al., 2022). GRACE (‐FO) observations have been validated against in situ ocean bottom pressure (Macrander et al., 2010), in situ groundwater (Li, Rodell, Sheffield, et al., 2019; Rateb et al., 2020), and regional and global hydrological modeling (Rateb et al., 2020; Zhang et al., 2017).

Yet, the missing solutions within the GRACE missions (22‐month) and between (July 2017–May 2018; 11 months) disrupt the continuity in the observations, reducing our ability to understand the evolution of the mass changes during these times and to perform relevant hindcasting. For the missing solutions within the missions, linear or spline interpolations are performed to fill these gaps, without proper justification, or constraining the uncertainties in these solutions. For the gap between the two missions, different approaches have been used to fill the missing data and reconstruct the gap, and can be summarized as follows: For the ice sheets, the independent mass balance changes were incorporated with GRACE (‐FO) observations to infer the status of the ice loss between 2017 and 2018 (Velicogna et al., 2020); For the land hydrology, where the most of the reconstruction studies focused on, the total water storage (TWS), which represents the sum of all storage compartments (snow water equivalent, surface water, soil water, groundwater, and ice) was reconstructed using Global Positioning Systems (GPS) (Rietbroek et al., 2014), or informed using hydroclimate predictors within statistical learning framework for the first mission (Li et al., 2020; Li, Wang, et al., 2019; Mo et al., 2022; Sun et al., 2021, 2020; Wang et al., 2021; Yang et al., 2021). Assimilating GRACE into land surface modeling (e.g., CLSM‐F2.5) is another approach to fill the gap in GRACE (‐FO) over land (Li, Rodell, Kumar, et al., 2019). Using low resolution gravity data from other satellite data (e.g., SWARM satellites), the global mass variability and missing solutions also can be retrieved (Forootan et al., 2020; Lück et al., 2018; Meyer et al., 2019; Richter et al., 2021). A recent study used the decomposed empirical orthogonal functions (EOF) modes of GRACE data to derive the temporal gravity field from Satellite Laser Ranging (SLR) data and extended GRACE‐like data back to 1992 (Löcher & Kusche, 2021). Alternatively, data‐driven approaches have been used (e.g., singular spectrum analysis (SSA) and its derivatives to infer the missing solutions for the trend and oscillatory changes (Wang et al., 2021; Yi & Sneeuw, 2021). The latter approach yielded deterministic outcomes and showed consistent results for spherical harmonics ≤30°, and low variance results for degrees >30 (Yi & Sneeuw, 2021).

The previous studies reflect one or more of the following conditions: (a) reliance on external information to impute missing solutions which compromise the uniqueness of GRACE (‐FO) signals. External information is either sparse and cannot apply to all mass change fields (e.g., GPS) or has its own biases and uncertainties (e.g., hydroclimate predictors), or is epistemically uncertain (e.g., lack of full parametrizations of the mass change data e.g., hydrological and mass balances models). Such biases and uncertainties can propagate into GRACE (‐FO) reconstructed data. For example, there is a lag between the hydroclimate predictors (e.g., sea surface temperature, air temperature, soil moisture, climate teleconnection), and the target variable (GRACE (‐FO) like data), and it varies by variable and across the regions. When this lag is not recognized or fixed across the variables or over the globe, the phase of the reconstructed data will shift and the amplitude can be underestimated or overestimated. However, still, there is value to include the external information to inform GRACE data on the climate‐driven variability historically (before 2002) with the proposed decaying function of the predictors (Humphrey & Gudmundsson, 2019). *S*tudies based on data‐driven approaches (Wang et al., 2021; Yi & Sneeuw, 2021) did not use external information to infer the gap between GRACE (‐FO) data. (b) In addition, when missing solutions are reconstructed, they are usually deterministic estimates (single values) without uncertainty quantification of the signal or its compartments (e.g., trend, interannual, annual, and semiannual). These challenges call for innovative, robust, and flexible approaches to infer the missing solutions which preserve the uniqueness of GRACE (‐FO) data, can apply to all the spheres of mass changes (land, ocean, and ice sheets), and provide estimates for the full distribution of the total observed and missing GRACE signal and its compartments.

**Figure 1:**
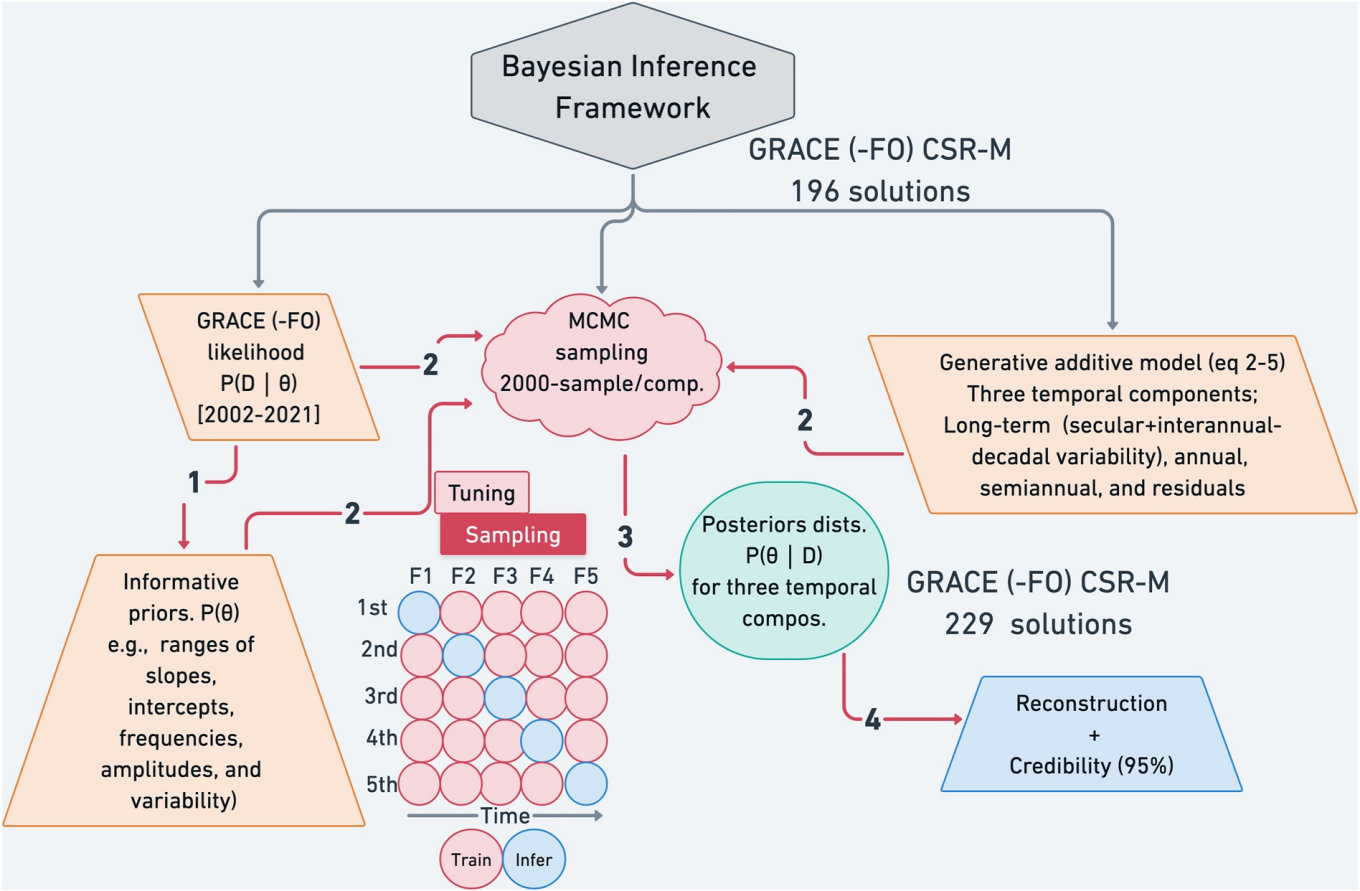
Flow chart of the method used to impute the missing solutions within and between Gravity Recovery and Climate Experiment and its Follow On (GRACE (‐FO)) missions. First, the informative priors were derived from the GRACE (‐FO) for a single grid point/basin time series as the ranges of the intercepts, slopes, variability, amplitudes, and frequencies of the annual and semiannual cycles in the GRACE (‐FO) time series, assuming an additive generative model describing the geophysical signal in GRACE data as long‐term variability (secular trend + interannual to decadal variability), annual, and semi‐annual. Second, we combined the likelihood data and the priors in the Markov Chain Monte Carlo sampling to generate posterior distributions for each of the component storages. Third, we merged the median of the posteriors for the component storages to reconstruct the full GRACE (‐FO) total water storage and its uncertainty at 95% credible interval. We added the residuals back to the observed time series to preserve the same variability as the original GRACE time series. We applied 5‐fold cross validations to validate the model internally and generate a predictive posterior distribution that can be used to infer the present and near future of the total signal.

The primary objective of this research was to reduce the limitations of previous approaches by adopting a Bayesian inference framework to decompose and model the signals in GRACE (‐FO) missions to provide a continuous reconstructed time series of GRACE (‐FO) data with detailed uncertainty quantification (Figure 1). Unique aspects of this research include:

1. Relying only on GRACE (‐FO) observations to impute missing solutions within and between the two GRACE missions.
2. Generating probability distributions of existing and missing solutions, for the signal compartments and reconstructing the full signal using posterior distributions, thus accounting for total uncertainties in the compartments and the full signal over the observed and missing times.
3. Inferring missing solutions over all spheres of mass change (e.g., land, ocean, ice sheets).

## 2 Materials and Methods

### 2.1 GRACE (‐FO) Data

The GRACE (‐FO) data were based on the University of Texas at Austin, Center for Space Research, release 06 version 02 mason solutions (CSR‐M RL06 V02) which extend from April 2002 through April 2021 (Save, 2020; Save et al., 2016). CSR‐M RL06 V02 mass change anomalies are relative to the 2004–2009 baseline. Standard corrections were applied to these solutions, including replacing C_20_ and C_30_ coefficients with SLR estimates (Loomis et al., 2019), optimizing geocenter motions C_21_ coefficients (Sun et al., 2016), and correcting glacial isostatic adjustment (GIA) using ICE6G‐D model (Richard Peltier et al., 2018). In CRS‐M RL06, the hexagonal tiles are split into two along the coastline to reduce leakage between the land and the ocean. CSR‐M RL06 is represented by 0.25° × 0.25° grid resolution, which is equivalent to a 1° × 1° geodesic grid at the equator. In our analysis, we restored the native resolution of the 1° × 1° grid to ease the computation time. These solutions are applicable to all mass changes (Save, 2020).

### 2.2 CLSM‐F2.5 Model

Total Water Storage (TWS) from the Global Land Data Assimilation System (GLDAS‐2.1) Catchment Land Surface Model (CLSM) model (Koster et al., 2000) was used to compare with the reconstructed GRACE TWS. The CLSM model was selected because simulated TWS values from CLSM are like those from GRACE (Girotto et al., 2016). CLSM‐TWS is the sum of plant canopy water, snow water equivalent, root‐zone soil water (0–100 cm), and unconfined and semi‐confined groundwater (Li, Rodell, Kumar, et al., 2019). CLSM‐TWS is limited by bedrock depth, which was expanded to 2‐m to accommodate the representation of the severe drought conditions in the southwestern U.S (Houborg et al., 2012). Groundwater storage in CLSM reflects only the model parameterizations of the bedrock depth and is calculated as the difference between the water profile storage and the water in the root zones as a groundwater anomaly, not total groundwater storage. The model does not simulate the surface water or biomass water and does not include human drivers of TWS (e.g., irrigation, reservoir impoundment). The snow water equivalent is represented by three layers of a snow model (Lynch‐Stieglitz, 1994) and no permanent ice or snow is simulated. GLDAS 2.1 is forced with combinations of the Agricultural Meteorological Model (AGRMET) and Global Data Assimilation (GDAS) radiation fields and disaggregated Global Precipitation Climatology Project (GPCP) 1° daily precipitation (Huffman et al., 2001). We use CLSM‐TWS estimates at a resolution of 1° × 1° grid for the period April 2002–April 2021.

### 2.3 Bayesian Modeling of GRACE (‐FO) Data

#### 2.3.1 Theory

To maintain continuity in GRACE (‐FO) observations in the present and infer missing solutions in the near future, we adopted a probabilistic approach to model mass change data from GRACE (‐FO) in terms of their intrinsic components. The idea is that the GRACE (‐FO) signal (e.g., annual cycle) parameters are random variables generated from distribution and are not unique. If we know such distributions given the existing GRACE (‐FO) data, we can generate sufficient sampling of the annual cycle over the missing and existing epochs. By ensuring that the sampling converges over the GRACE (‐FO) period, we can infer the annual cycle during the missing periods using these samples and obtain uncertainty over the whole period. Such estimates can be used to infer the present and future distributions of the signal and can be updated when new observations are made available.

In a Bayesian framework, the GRACE (‐FO) data (D) and the temporal components parameters (*θ*) can be described as follows:

(1) $$P\left(\theta |D\right)=\frac{P\left(D|\theta \right)\times P\left(\theta \right)}{\sum P\left(D|\theta \right)\times P\left(\theta \right)}$$

where P(D|θ) is the probability of observing GRACE (‐FO) data (likelihood), given the temporal component parameter θ; P(θ) is the prior distribution of the parameters; and P(θ|D) is the posterior distribution of θ. The denominator is the sum of the likelihoods, given the priors. Finding the posterior distribution is not feasible without knowing the explicit forms of the prior and likelihood. A sampling‐based method, such as Markov Chain Monte Carlo (MCMC), is often used to circumvent this problem. In the context of the current study, we incorporate the prior knowledge of the distributions of the long‐term variability which includes secular trend and interannual‐decadal variations (intercepts, and slopes) as t‐distribution to account for the flat tails, and Gaussian distribution for annual and semi‐annual components. We then used the MCMC method to generate 2,000 samples from the posterior distribution (*P*(*θ*|*D*) for each component (Durbin & Koopman, 2012; Harvey, 1990; Scott & Varian, 2014) (Figure 1). The observational equation for the GRACE (‐FO) data can be described as:

(2) $$Y_{t}^{\text{GRACE}\;\left(‐\text{FO}\right)}=\mu_{t}+\delta_{t}+\gamma_{jt}\cos\left(\lambda_{j}\right)+\gamma_{jt}^{*}\sin\left(\lambda_{j}\right)+\epsilon_{t,\;}\;t=1,\text{…}.n$$

where $\mu_{t}$ is the intercept, $\delta_{t}$ is the slope, $\gamma_{jt},{\gamma_{jt}^{*}}_{,}$ are the annual or semi‐annual amplitudes for cos, and sin, respectively, $\epsilon_{t\;}$ is the residual, t is the time, $\lambda_{j}=2\pi j/S$, where *S* is the frequency of the annual (12‐month) or semi‐annual (6 months) signals. In a probabilistic framework, these components are stochastic and vary randomly over time and their coefficients drift according to random walk as.

For intercepts

(3) $$\mu_{t+1}=\mu_{t}+\delta_{t}+\epsilon_{t,\;}\;\epsilon_{t}∼{Τ}_{v_{\mu }}\;\left(0,\;\sigma_{µ}\right),\;$$

For slope

(4) $$\delta_{t+1}=\delta_{t}+\eta_{t\;}\;\eta_{t\;}∼{Τ}_{v_{\delta }}\;\left(0,\;\sigma_{\delta }\right),\;$$

For annual or semi annual

(5) $$\;\gamma_{jt+1}=\gamma_{jt}\cos\left(\lambda_{j}\right)+\gamma_{jt}^{*}\sin\left(\lambda_{j}\right)+\epsilon_{0t}\;\epsilon_{0t}∼N\;\left(0,\;\sigma \right)\;$$

(6) $$\gamma_{jt+1}^{*}=\gamma_{jt}^{*}\cos\left(\lambda_{j}\right)-\gamma_{jt}\sin\left(\lambda_{j}\right)+\epsilon_{1}.\;$$

where $\eta_{t\;}$ is an independent random variable with zero mean and standard deviation$\;\sigma_{\eta }$. Both $\epsilon_{t}\;\text{and}\;\eta_{t\;}$ are mutually independent and from $\delta_{t}$. $v_{\mu }$ is the level tail thickness, $v_{\delta }$ is the slope tail thickness. When $\eta_{t}=0,$ the trend is exactly deterministic linear, except for a noise model. However, since $\eta_{t\;}$ is vary normally, we call this component here a “long‐term”, which represents both the linear and non‐linear (interannual‐decadal) fluctuations. $\epsilon_{0},\;\epsilon_{1}$ are independent random variables with the same variance. For semi‐annual we repeat the trigonometric part in Equation 2 using different frequencies (6 months), with different ($\gamma_{jt},\gamma_{jt}^{*}$) amplitudes. ε is the residual shared among all components. In practice, we use a three‐layer model; long‐term variability, annual cycle, semi‐annual cycle, and model components simultaneously within the Bayesian computational framework.

#### 2.3.2 Inference

**Figure 2:**
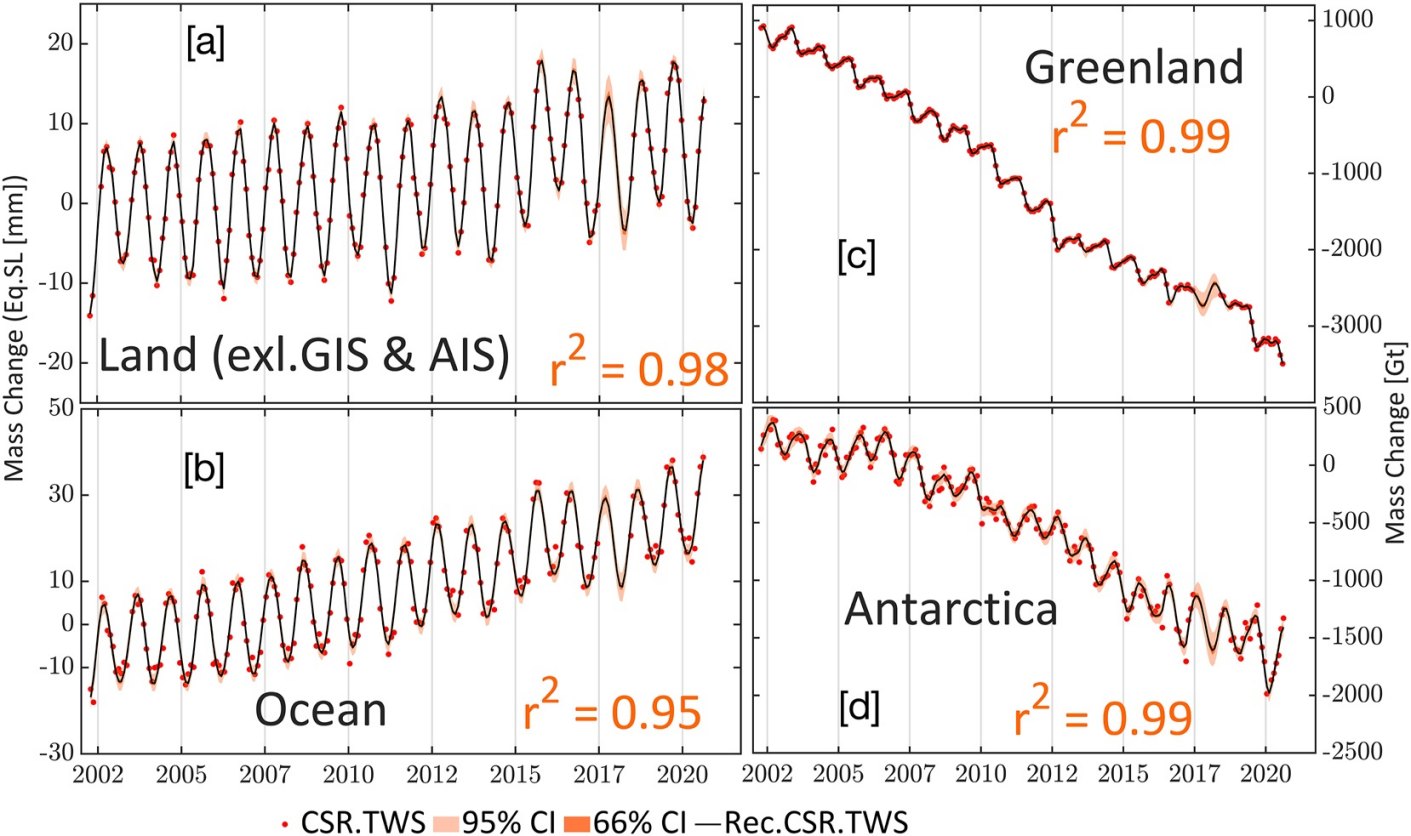
Mass change in three spheres, [a] land (excluding Greenland Ice Sheet (GIS) and Antarctic ice sheet (AIS)), [b] Ocean, expressed as equivalent sea level in mm, and Ice sheets ([c] Greenland and [d] Antarctica). The black line is the sum of the median posterior distributions for long‐term variability and annual and semi‐annual signals. Residuals were added back to periods with existing Gravity Recovery and Climate Experiment observations. The resorted solutions are represented by the reconstructed signal only without accounting for the residuals. Credibility of estimates is outlined with two probability levels (66%; likely and 95%; extremely likely).

**Figure 3:**
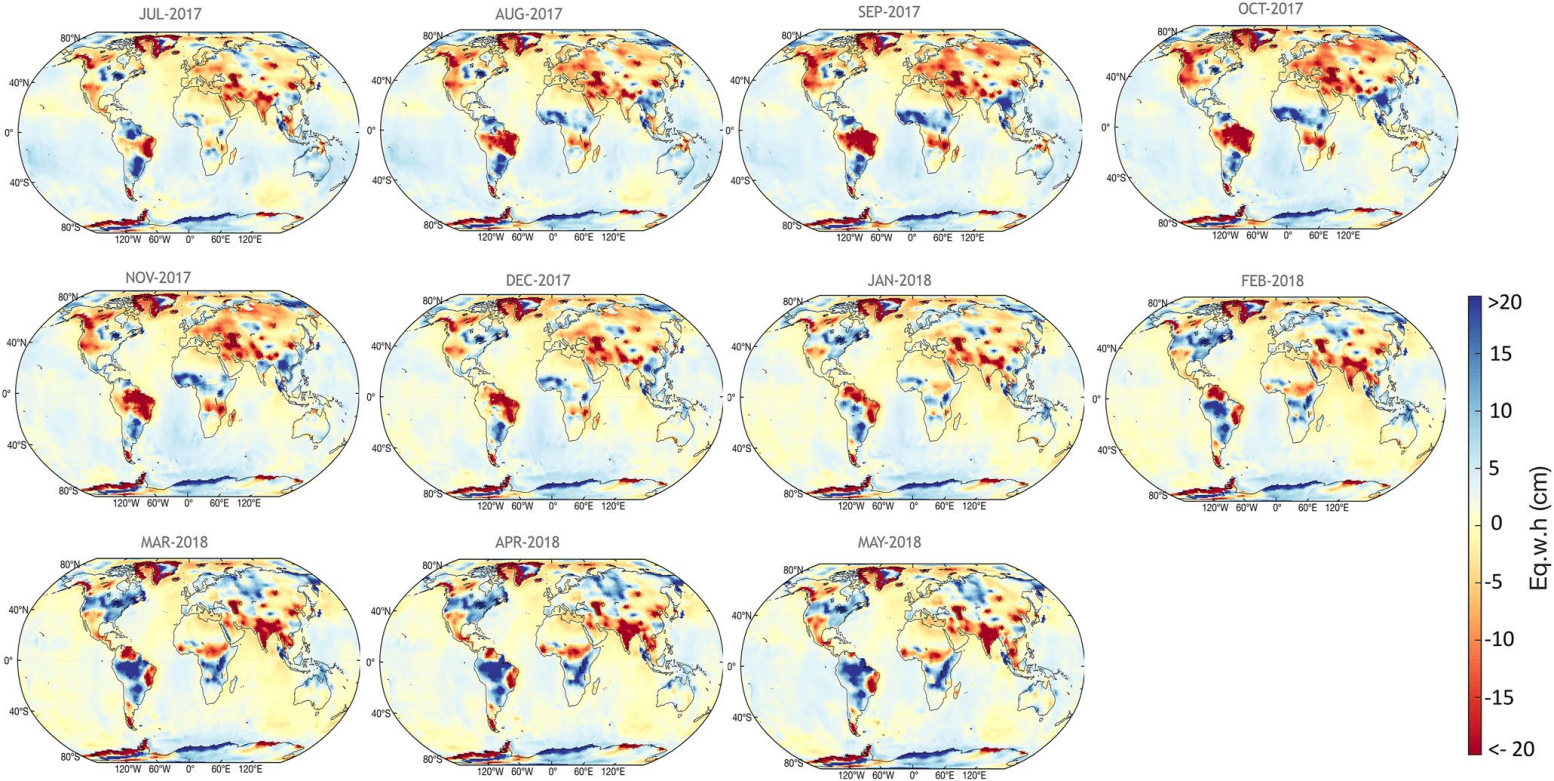
Modeled total water storage during the Gravity Recovery and Climate Experiment (GRACE) and GRACE‐Follow On gap as the sum of the median posterior distribution of long‐term variability (variability ≥12‐month, including secular trend and interannual‐decadal variations), annual and semi‐annual signals between April 2002 and April 2021, sampled from 2,000 steps using Markov Chain Monte Carlo with the No‐U‐Turn Sampling method. Uncertainties associated with these signals are provided in supplementary materials at 5% and 95% levels (Figures S2, S3 in Supporting Information S1).

**Figure 4:**
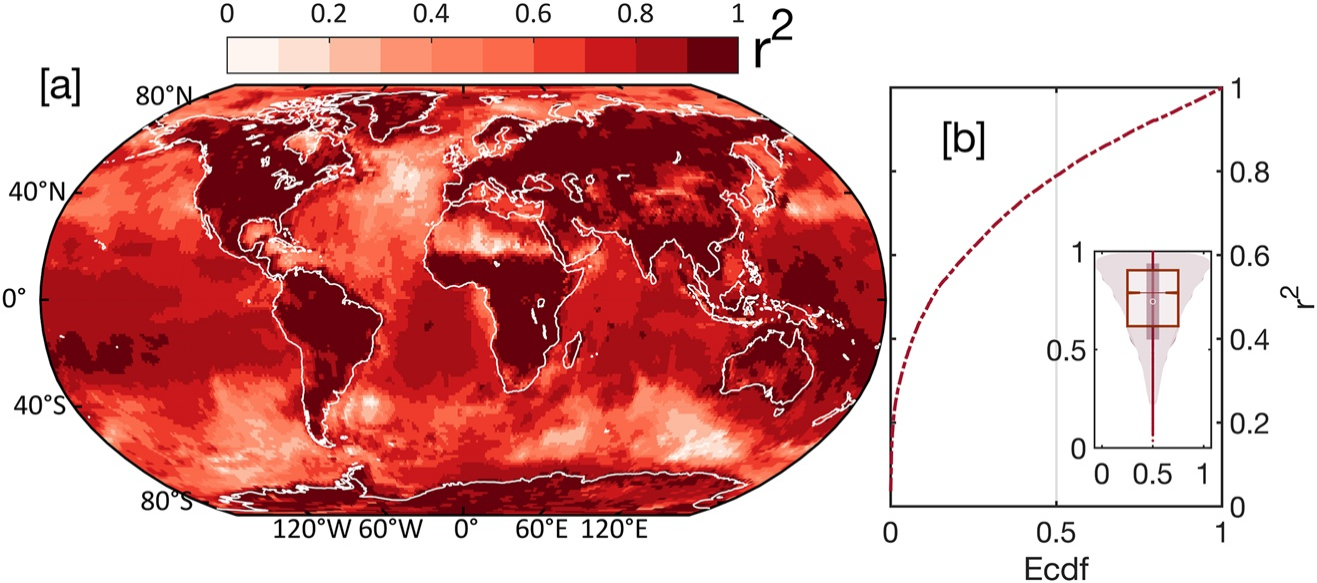
[a] Markov Chain Monte Carlo regression model diagnostic test with coefficient of determination (r^2^). [b] Empirical cumulative density function (ecdf) for r^2^ showing ≥80% of the grid cells have *r* ^2^ ≥ 58%. Variabilities in the predictable signal and the residuals are provided in SI (Figure S5 in Supporting Information S1).

We used four independent Markov chains to develop sample distributions using No‐U‐Turn Sampling (NUTS) for the components in GRACE (‐FO) data. Each chain starts with 2,000 warm ups (burn‐in) to generate an equilibrium distribution of sampling, followed by 2,000 samples to generate the posterior distribution for each component. Equilibration and MCMC convergence are achieved by ensuring that $\hat{R}$ ≤ 1.05 (Vehtari et al., 2021). $\hat{R}$ is a convergence diagnostic test that compares the variance within and between chains, with $\hat{R}$ close to unity indicating better convergence of the component parameters. There are no assumptions on the posterior distribution required by the MCMC approach. The MCMC approach generates solutions with high variance and low bias (Plummer et al., 2006). Generating these posterior distributions allows us to quantify uncertainties in the component parameters and test the probability of a given parameter. From each posterior distribution, we calculated the median and its credibility at 66% (likely) and 95% (extremely likely) probabilities for 30 hydrologic basins and land, ocean, and ice sheet global spheres. For the one‐degree gridded data, we limited uncertainties to 95%. Note this credible interval is a direct measure of the probability of the real (unknown) parameter given the existing observations and the prior distributions and differs from the known confidence interval. Posterior distribution medians are merged to represent the total signal. The residual component is added over the existing observations; thus, estimates over the missing times are the sum of the modeled components (Figures 2 and 3). The computation was carried out using Stan probablistic programming (Carpenter et al., 2017), using R interface (Team, 2013). An example of decomposing and modeling the GRACE (‐FO) data is given in supplementary materials (S1) (Figure S1 in Supporting Information S1). The method was applied to gridded data at a one‐degree grid‐scale (Figure 3), global mass change spheres time series (Figure 2), and 30 global hydrological basins (Figure S6, Table S1 in Supporting Information S1). The performance of the modeling and decomposition was evaluated using coefficients of determination (*r* ^2^) (Figure 4) and measuring the variability of the models and residuals (Figure S5 in Supporting Information S1). The reconstructed solutions over land were compared with output from the CLSM‐TWS model using four statistical metrics: correlation coefficients (*CC*) (Pearson, 1895), Nash–Sutcliffe Efficiency (NSE) (Nash & Sutcliffe, 1970), normalized root mean square error (NRMSE), and the ratio of standard deviations (rSTD).

#### 2.3.3 Model Criticism

To evaluate the performance of the Bayesian model and given the shortness of GRACE (‐FO) data, we partitioned the GRACE (‐FO) into 5 folds (Figure 1). Each fold comprises training and testing sets. In each fold, part of the data is decomposed as mentioned above and the posterior distributions are used to calculate predictive distributions for a year ahead. Then, statistical matrices (e.g., mean absolute error (MAE), root‐mean‐square error (RMSE), and coefficient of determination (r^2^)) are used to evaluate model predictions with the unseen testing part. We used 5 years as testing samples; the years with no missing values in GRACE (‐FO) (e.g., 2007, 2008, 2009, 2019, and 2020).

The model performance is measured by averaging the computed values for the five folds. The k‐fold cross validation of the reconstructed data was performed over 30 basins only to reduce the computational requirements with more data made available up to September 2021. In the final step, we trained the model with the entire reconstructed data and inferred once a year ahead (September 2022). We inferred the predictive posterior distributions (2000‐sample) and generated the median and 95% of a credible interval of TWSA predictions for the 30 basins (Figure S6 in Supporting Information S1). The predictive posterior distributions can be seen as forecasting for the present and can be augmented in present day applications (e.g., data assimilation) to overcome the latency of GRACE data.

#### 2.3.4 Model Diagnostics

To test the generated data independently, we evaluated the data over land against the CLSM‐F2.5 model output. We measured the following matrices.

(7) $$\;r^{2}=1-\frac{SS_{Residuals}}{SS_{Total\;}}\;$$

(8) $$CC=\;\frac{\sum_{i=1}^{N}\left(CLSM_{i}-\bar{CLSM}\right)\left(GRACE_{i}-\bar{GRACE}\right)}{\sqrt{\sum_{i=1}^{N}{\left({\text{CLSM}}_{i}-\bar{\text{CLSM}}\right)}^{2}}\sqrt{\sum_{i=1}^{N}{\left({\text{GRACE}}_{i}-\bar{\text{GRACE}}\right)}^{2}}}\;$$

(9) $$NSE=1-\frac{\sum_{i=1}^{N}{\left({\text{GRACE}}_{i}-{\text{CLSM}}_{i}\right)}^{2}}{\sum_{i=1}^{N}{\left({\text{CLSM}}_{i}-\bar{\text{CLSM}}\right)}^{2}}$$

(10) $$\text{NRMSE}=100\times \frac{\sqrt{\frac{1}{N}\sum_{i=1}^{N}{\left({\text{GRACE}}_{i}-{\text{CLSM}}_{i}\right)}^{2}\;}}{{\text{CLSM}}_{\text{max}}-{\text{CLSM}}_{\text{min}}},\;$$

where SS is variance, i is time step, N is number of months, and $\bar{\text{GRACE}}\;\text{and}\;\bar{\text{CLSM}}$ are means of GRACE (‐FO) ‐TWS and CSLM‐TWS, respectively.

## 3 Results and Discussion

### 3.1 Model Validation and Performance

We evaluated the reconstructed data using three scenarios. First using k‐fold validations for 30 basins, we found a median *r* ^2^ = 0.98 for the basins, indicating higher performance over different parts of the data (Table S1 in Supporting Information S1). Similarly, the measured RMSE median = 50.6 km^3^. These matrices were calculated as the mean of the five folds of the data. The predictive posterior distribution is then generated after training the data for the entire reconstruction period for these basins (04/2002‐04/2021), and the distributions of the signal were inferred for a year ahead (09/2021‐09/2022). The distributions are represented by the 2,000 samples, the median, and the 95% credible interval (Figure S6 in Supporting Information S1).

Second, using model outputs against the original data, we found the model fits the data well for the land, ocean, and ice sheets (*r* ^2^ ≥ 0.95), suggesting that the model captures most of the variability in the original data. The remaining 5% of the variance was restored by adding the residual back into the models to preserve the variability of the original data. In hydrologic basins, r^2^ for a median model fit = 0.99 (Figure S4, Table S1 in Supporting Information S1). At one‐degree grid scale, the r^2^ for model fit is ≥0.58 for ∼80% of the grid points (Figures 4a and 4b). Areas with low r^2^ (≤0.5) represent ∼20% of Earth's surface and include the Sahara Desert, North Atlantic Ocean, southern Indian, and Pacific Oceans, and the eastern Arctic Ocean, where mass changes are small. The Sahara Desert is an arid to hyper‐arid zone, with minimal precipitation (Nicholson et al., 2012) and TWS changes are driven mostly by a trend component caused by groundwater depletion (Frappart, 2020; Rateb et al., 2017; Scanlon et al., 2022). Inspection of the Tamanrasett Basin in northwest Africa (semiarid to arid climate) reveals a wide range of uncertainty in the reconstructed model and lower r^2^ (0.41) and poor performance relative to the CLSM model (Figure S4, Table S1 in Supporting Information S1). Seasonal mass changes in the Sahara Desert resemble noise levels in the GRACE solutions (Boy et al., 2012). Low r^2^ in the southeastern Pacific and Indian Oceans and North Atlantic may result from low mass change at semi‐annual and annual scales and high mass changes at synoptic scales (shorter than semi annual) that could not be captured by the models. Using daily swath CSR GRACE data, Bonin and Save (2020) show that these areas in the southern Indian, Pacific, and North Atlantic oceans have high variability in the sub‐monthly signal (40–50 mm water height). Daily variability in water flux ($\frac{\text{dTWS}}{\text{dt}}$) in these regions is ∼5–8 mm/day, estimated using daily ITSG‐2018 GRACE solutions (Eicker et al., 2020). In our generative model, the high‐frequency signal (e.g., synoptic variations) are not modeled given their weakness globally and the inadequacy of the monthly data to capture them; therefore, they are left as a part of the residuals. However, because we add back the residuals to the reconstructed signal, these variations are preserved in the reconstructed data.

### 3.2 Comparison With CLSM Model

**Figure 5:**
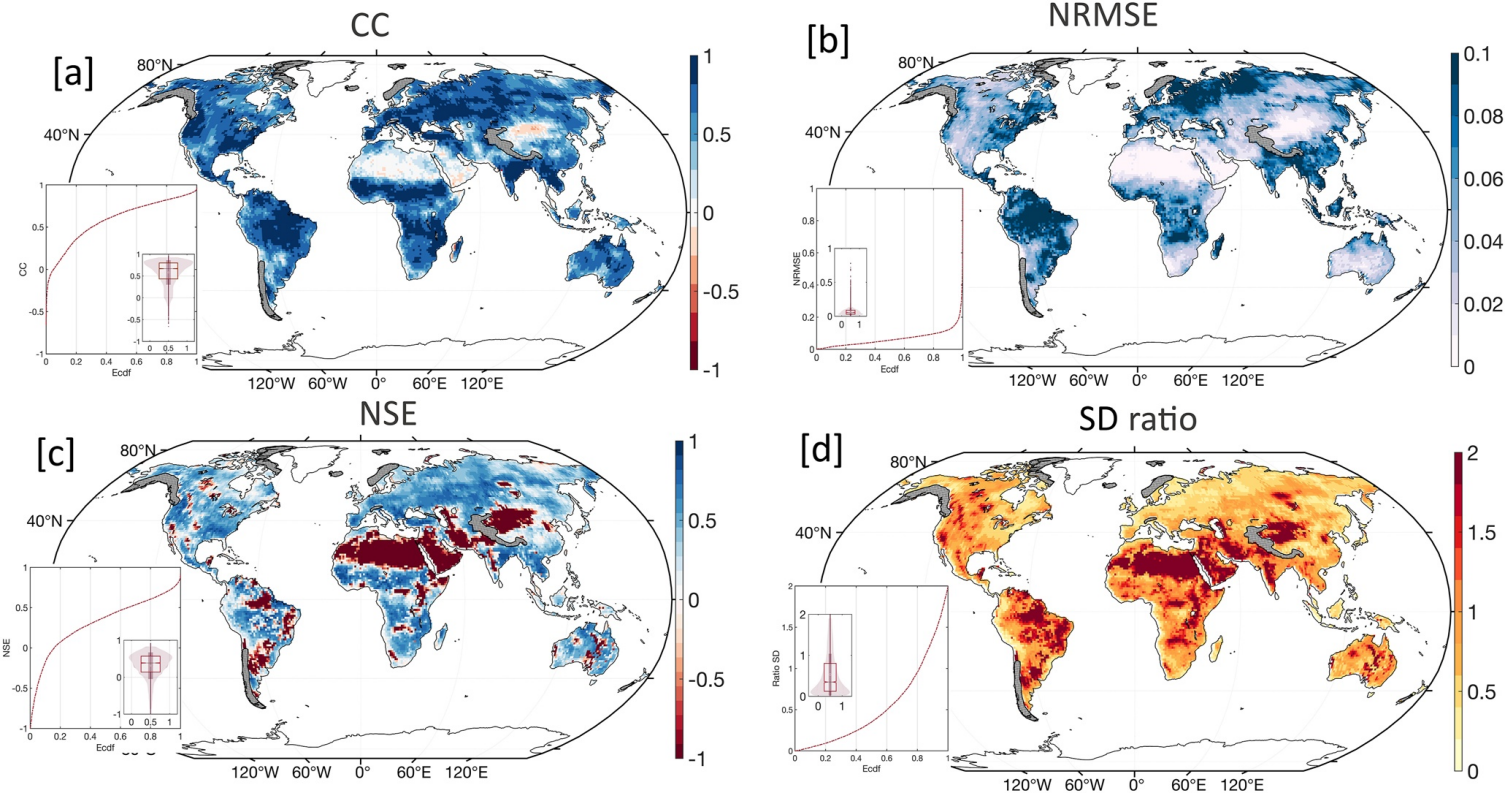
Four evaluation tests of Gravity Recovery and Climate Experiment (GRACE) and GRACE‐Follow On mass change reconstructed data over land with catchment land surface model (CLSM)‐total water storage between the April 2002 and April 2021 and the associated ecdf. [a] correlation coefficient, [b] normalized mean square error, [c] Nash–Sutcliffe Efficiency, and [d] ratio of variability. Results are hachured over 11 land glaciers for consistency because the CLSM model does not simulate permanent snow or ice.

Third, reconstructed GRACE (‐FO) model outputs were compared with the CLSM model (TWS) over land at grid scale (Figure 5) and in hydrological basins (Table S1 in Supporting Information S1), after removing the secular trend component. The trend component results primarily from human impacts on groundwater storage (e.g., Sahara desert, High Plains, Central Valley, and North China Plain) or from a change in the land use (e.g., west Africa) (Rodell et al., 2018). Such drivers of TWS are not considered in the CLSM model. Therefore, we compared the detrended TWS in the GRACE (‐FO) reconstructed model and CLSM TWS between 2002 and 2021. Phase agreement is high (CC > 0.5) for 60% of the grid points, with a median of 0.68 (Figure 5a). Median correlations increase to 0.88 for the hydrologic basins (Table S1 in Supporting Information S1). Areas with low CC are found in the Sahara desert and North China. NSE test results are similar to CC, where positive values (NSE ≥ 0.0) are found in ≥80% of the grid points, showing similar predictive skills in the reconstructed GRACE (‐FO) TWS and CLSM‐TWS. For the basins, the NSE median is high (0.7) showing the good performance (Table S1 in Supporting Information S1). Negative and relatively small positive values ≤1 were found in the Sahara desert, Arabian Peninsula, southern High Plains, northern and central Argentina, eastern Brazil, Iran, and northern China. The NRMSE, reflecting the portion of the reconstructed GRACE (‐FO) TWS that is resolved by the CLSM TWS, shows higher values over humid regions in the tropics, high latitudes, and relatively low values over arid areas in North Africa, North China, and Australia, indicating general agreement and closer variance in humid regions and larger differences in the arid areas. The ratio of variability (rSTD) between the reconstructed GRACE (‐FO) TWS and CLSM model shows that the variability in the GRACE (‐FO) TWS is two times that of CLSM‐TWS over these arid areas. This ratio is near unity (median 0.99) for the hydrologic basins, which suggests greater consistency between the two datasets at the basin scale. The CLSM model and land surface models in general show poor performance in arid regions relative to GRACE (‐FO) in terms of water balance change even with averaging of these models (Rateb et al., 2019). Averaging these models improves agreement in terms of correlations but does not attain the variability of GRACE‐TWS (Rateb et al., 2019). Inspecting CLSM performance relative to GRACE during the 2002–2017 period shows that CLSM has a negative trend in surface water basins (e.g., Congo, Niger, Parana), and deviates from GRACE (‐FO) after 2012. The cause of these deviations is beyond this study. To conclude, a comparison between the reconstructed GRACE (‐FO) signal and CLSM‐TWS over land between 2002 and 2021 shows good agreement at the grid scale and during gap periods, except in the regions where the CLSM‐model performs poorly (e.g., arid regions, etc). Agreement and consistency between GRACE and CLSM are higher at the basin scale.

### 3.3 Comparison With Other Reconstructed Data

**Figure 6:**
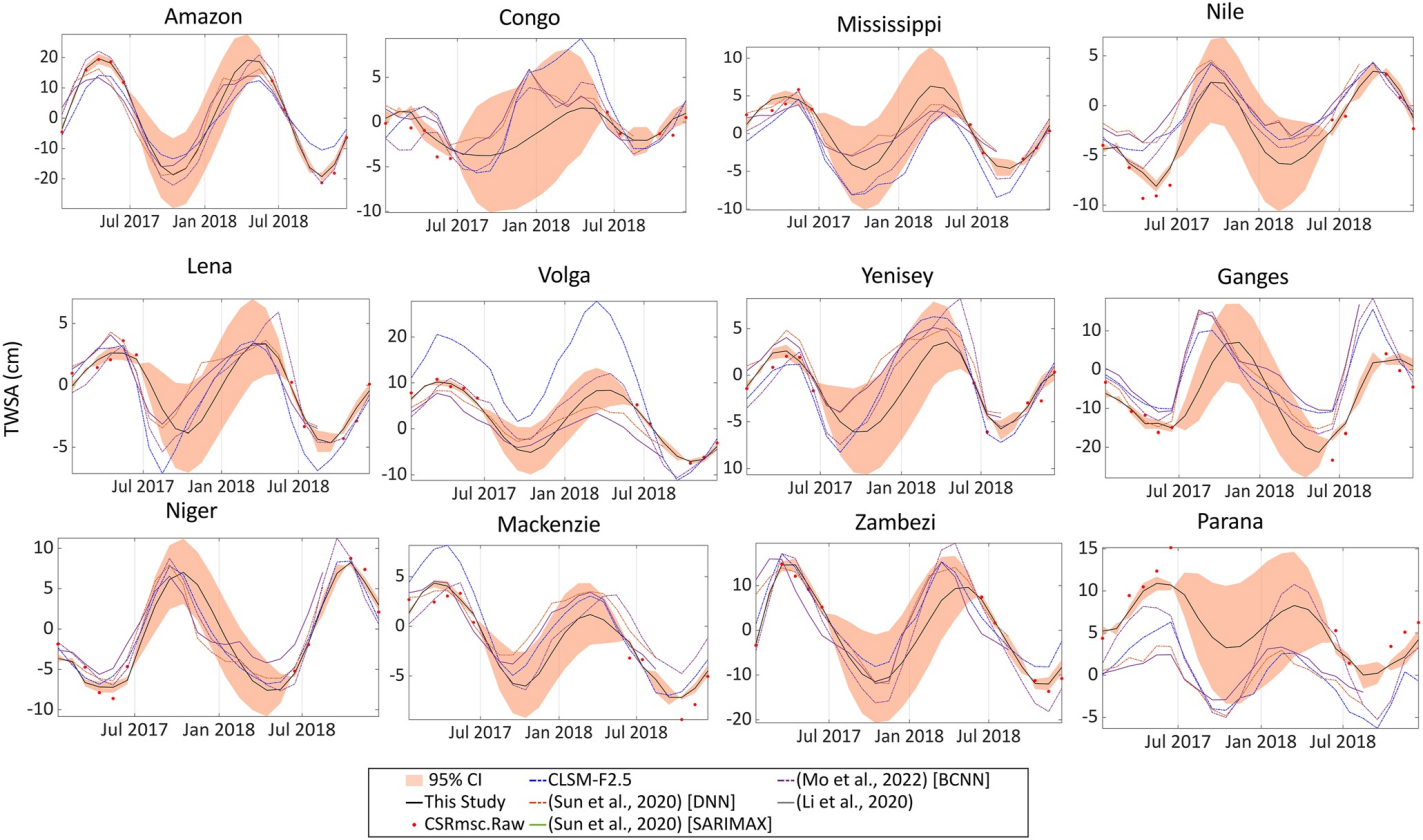
Time series for the five reconstructed data from four studies for the gap period between Gravity Recovery and Climate Experiment (GRACE) and GRACE‐Follow On missions. The original data from the two missions are shown in red circles. Results from this study are plotted in black lines with the 95% of credible interval (light orange).

We compared our reconstructed data with data from four other GRACE reconstructions (Li et al., 2020; Mo et al., 2022; Sun et al., 2020). These studies were concerned with filling the gap between the missions only. We provide an example of the comparison in Figure 6 and summarized the results in Figure S8 in Supporting Information S1. The comparison also includes the de‐trended TWSA from the CLSM model, and raw CSR‐M GRACE (‐FO). Data from Li et al. (2020) are only available for 37 basins and Sun et al. (2020) provided two reconstruction results based on Deep neural network (DNN) and seasonal autoregressive integrated moving average with exogenous variables (SARIMAX) models. Data shown in Figure 6 are for 24 months from January 2017 to December 2018 to show a level of consistency across the reconstructed data and the real data from GRACE (‐FO) missions. In general, the results show a good agreement across these reconstructed data in capturing missing signals during the gap. This signal is mostly dominated by the annual cycle. The median NSE of our reconstruction is 0.40, ranging from −0.5 to 0.6. Our reconstruction shows a median accuracy error of 44 mm and other studies have a range from 46 to 82 mm relative to the CLSM model. Comparing these results with the raw GRACE (‐FO) data shows our reconstruction is the closest to the raw data in terms of timing and magnitude (Jan‐Jun 2017; Jun‐Dec 2018). One striking difference between our reconstruction and all other data is that in some basins, the other data are phases shifted and underestimate or overestimate the magnitude of TWS relative to the original data and our reconstructed data (e.g., Congo, Ganges, Lena, Yenisey). Such differences can be attributed to the fixing the lags across the hydroclimate variables (e.g., sea surface temperature, air temperature, precipitation, soil moisture, evapotranspiration, TWS (reanalysis data), TWS (land surface modeling), and climate teleconnection indices) or across the globe in these other studies. These variables are used to learn GRACE‐like data from the first mission, and then forecast the gap. While our reconstruction relies only on sampling for each signal component and makes use of all GRACE (‐FO) data. Our results agree with the learning methods that incorporated hydroclimatic data to reconstruct the gap in terms of CC and NSE and outperform them in term of RMSE. Specifically, the results are more consistent with DNN and Bayesian CNN (BCNN) reconstructions (Mo et al., 2022; Sun et al., 2020), than SWARM driven, statistical learning driven (ANN, ARX, MLR) outputs from (Li et al., 2020). The training periods are similar in both studies (04/2002‐01‐06/2014) (Mo et al., 2022; Sun et al., 2020), but the testing period of performance is different: 04/2014 to 06/2017 (Sun et al., 2020), and 04/2014‐06/2017, and 01/2018‐09/2020 (Mo et al., 2022).

To summarize, while the previous studies were concerned only with bridging the gap between the two missions (11 months) and relied on the hydroclimatic predictions to inform GRACE predictions, this research was interested in generating a distribution over the existing and all the33 missing solutions from the perspective of the data‐generation geophysical processes. Hence, it provides a full uncertainty over the entire GRACE (‐FO) mission time series, regarding the CSR‐M solutions.

Given the probabilistic outcomes of this approach, the predictive distributions of the signal can be inferred in the present or the near future (Figure S6 in Supporting Information S1) without incorporating external data. Also, the approach can ingest the new GRACE (‐FO) data when made available. This flexibility has an advantage over other deterministic learning methods, to inform the near real time GRACE (‐FO) applications (e.g., data assimilations).

## 4 Conclusions

In this research, missing solutions within and between the two GRACE missions (33 solutions) were inferred using a probabilistic framework. We modeled GRACE (‐FO) data (196 solutions; 04/2002–04/2021) by decomposing the geophysical signal into temporal components; long‐term (secular trend + interannual to decadal variability), annual, and semi‐annual). We used informative priors on the components (e.g., ranges of intercept, slopes, amplitudes, frequencies) and inferred the distribution of these components using the Markov Chain Monte Carlo sampling method. We generated 2000 samples as a posterior distribution of each component and reconstructed the complete GRACE (‐FO) data (229 solutions between 04/2002 and 04/2021) by adding medians of the posterior distributions of the temporal components and quantifying uncertainty at a 95% level. We obtained results over a one‐degree grid scale and over hydrologic basins. The reconstructed data explain the variability in GRACE (‐FO) data (median r^2^ 99%) at the hydrological basin scale and greater than 60% for ≥70% of the grid points. Low model performance was found in the Sahara Desert, Southeastern Indian and Pacific oceans, and North Atlantic Ocean. These areas either have low mass change at annual/semi‐annual timescales and long‐term variability or the meaningful signal was captured as part of the residuals as synoptic variations (e.g., southern oceans areas). The results are consistent with other methods that incorporated hydroclimate indicators and outperform them in terms of accuracy relative to original data and land surface modeling. Our method further provides a distribution of the missing and the existing observations from the perspective of the data generation processes, thus it provides total uncertainty over the GRACE mission's data. Given the probabilistic outcomes of this method, we generated a predictive distribution of the unseen signal (up to September 2022) based on internally tested data. These distributions can be ingested in near real‐time applications (e.g., data assimilations) to overcome GRACE data latency from the science data centers. Bayesian modeling of GRACE data is a data‐driven flexible approach to model the GRACE (‐FO) data and infer uncertainties over the existing and missing solutions from the perspective of the data‐generation processes and does not require external information.

## Conflict of Interest

The authors declare no conflicts of interest relevant to this study.

## Supporting information

Supporting Information S1

## Data Availability

CSR GRACE/GRACE‐FO RL06 Mascon Solutions are accessible through (GRACE/GRACE-FO%20-%20Gravity%20Recovery%20and%20Climate%20Experiment%20(utexas.edu). The reconstructed data are hosted by the Texas data repository and freely available at (https://doi.org/10.18738/T8/5MPOJU) (Rateb, 2021). Data are stored in NetCDF format and contain reconstructed GRACE (‐FO) that is based on CSR mascon and its uncertainty.
