# Supplementary material for: Reconstruction of GRACE Mass Change Time Series Using a Bayesian Framework
Source: Earth Space Sci. 2022 Jul 7;9(7):e2021EA002162. doi: 10.1029/2021EA002162 (PMC9400854; doi:10.1029/2021EA002162)
Supplement: Supplementary file 1 — Supporting Information S1 [file ESS2-9-e2021EA002162-s001.docx]

**Reconstruction of GRACE Mass Change Time Series Using a Bayesian Framework**

Ashraf Rateb^1^, Alexander Sun^1^, Bridget R. Scanlon^1^, Himanshu Save^2^, Emad Hasan^2^

^1^Bureau of Economic Geology, The University of Texas at Austin, Austin, 78758, TX, USA

^2^Center for Space Research, The University of Texas at Austin, Austin, 78758, TX, USA

*Correspondence to*: Ashraf Rateb ([ashraf.rateb@beg.utexas.edu](mailto:ashraf.rateb@beg.utexas.edu))

Nine Figures

One Table

**List of Figures**

[Figure S1. An example of decomposing and modeling the GRACE (FO). [a] original GRACE and GRACE-FO observations with missing observations (33 solutions as represented by the lines discontinuity). [b] decomposing the long-term variability, which is the sum of the secular trend and interannual cycle (blue line is the median posterior, bisque lines are a 2000 sample from the posterior for this component. [c, d, e] same as b but for annual, semiannual cycles, and residuals. 4](#_Toc106018152)

[Figure S2. Lower bound of credibility (5%) of the reconstructed signal during GRACE and GRACE-FO gap obtained after joining the median of posterior distributions of the temporal components. 5](#_Toc106018153)

[Figure S3. Upper bound of credibility (95%) of the reconstructed signal during GRACE and GRACE-FO gap obtained after joining the median of posterior distributions of the temporal components. 6](#_Toc106018154)

[Figure S4. Total Water Storage (TWS) anomalies simulated by CLSM-F2.5 model during GRACE and GRACE-FO gap 7](#_Toc106018155)

[Figure S5. The variability of the predicted signal and the residuals in cm unit. The predicted values are the fit of long-term variability, annual and semiannual with 2000 model for each component. 8](#_Toc106018156)

[Figure S6. An example of the reconstructed GRACE like data represented by four examples (Amazon, Congo, Mississippi, Parana, Niger and Yangtze. The reconstructed data are in black lines (04/2002 to 09/2021), original time series are in red circles from CSR.M. Predictive posterior distributions of (4000) samples are in grey lines for the period (09/2021- 09/2022), with their median in blue lines, and 95% credible interval (dashed green lines) (a-f). The performance of the K-fold cross validations is summarized by r-square and RMSE tests (g-h) for 30 basins. 9](#_Toc106018157)

[Figure S7. Examples of filling the missing observation within and between GRACE missions for 12 hydrological basins. Red points are the original observations as calculated by the CSR mascon solutions; the black line is the reconstructed signal obtained after joining the median of the posterior of the temporal components (Figure S1), with two-level of credibility (66%, 95%), and the blue line is CLSM-TWS. 10](#_Toc106018158)

[Figure S8. Performance of the four reconstructed data for the global basins (186 basins , figure S9),  relative to this study and the CLSM model (de-trended data). The results for Li et al. [2020] are available for the 37 basins only. 12](#_Toc106018159)

[Figure S9. Location of 186 hydrological basins outlined used to compare the reconstructions data from four studies (Figure S8) 13](#_Toc106018160)

**List of Tables.**

[Table S1.  Model diagnostic tests for the reconstructed data using Coefficient of determinations (r^2^) for 30 hydrological basins. The comparison with the CLSM-TWS was measured for the whole period between 04/2002 and 08/2020 using four tests; NSE is the (Nash Sutcliff Efficiency), RMSE (Root Mean Square Errors), Std (Standard Deviations), and CC (Correlation Coefficient). 11](#_Toc99359038)


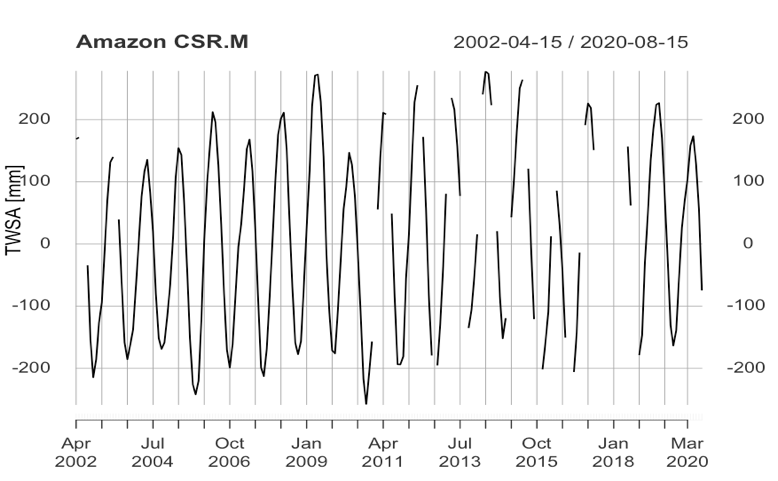

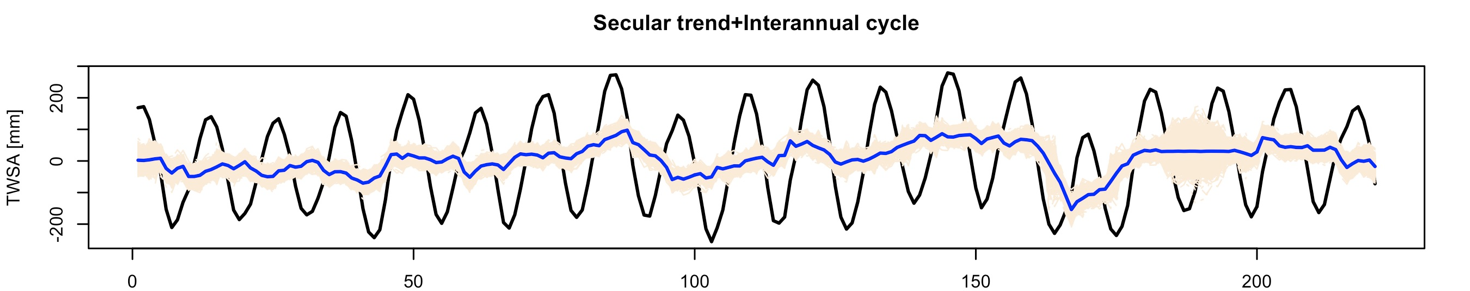

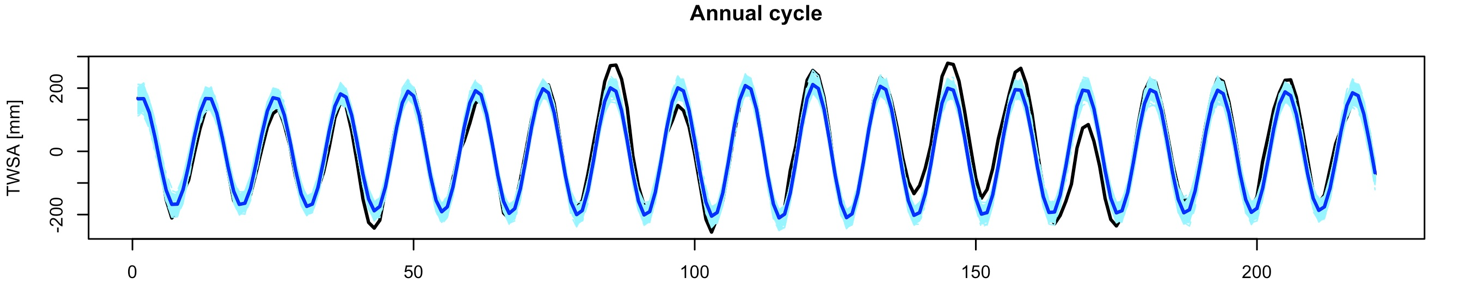

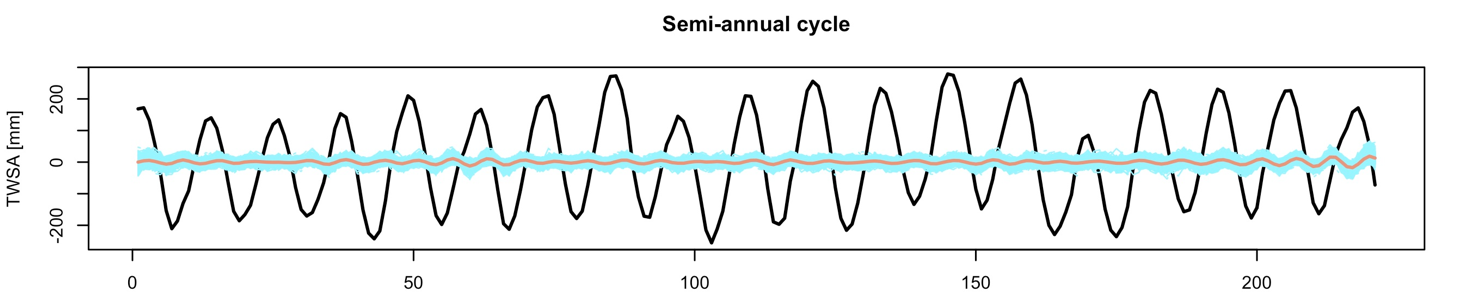

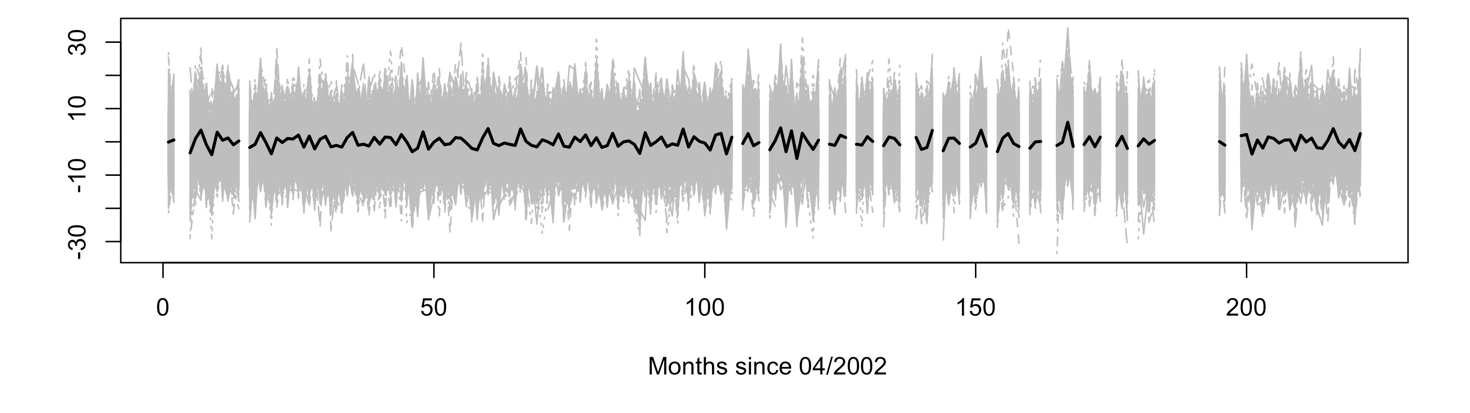


[a]

[b]

[c]

[d]

[e]

Figure S1. An example of decomposing and modeling the GRACE (FO). [a] original GRACE and GRACE-FO observations with missing observations (33 solutions as represented by the lines discontinuity). [b] decomposing the long-term variability, which is the sum of the secular trend and interannual cycle (blue line is the median posterior, bisque lines are a 2000 sample from the posterior for this component. [c, d, e] same as b but for annual, semiannual cycles, and residuals.





Figure S2. Lower bound of credibility (5%) of the reconstructed signal during GRACE and GRACE-FO gap obtained after joining the median of posterior distributions of the temporal components.





Figure S3. Upper bound of credibility (95%) of the reconstructed signal during GRACE and GRACE-FO gap obtained after joining the median of posterior distributions of the temporal components.


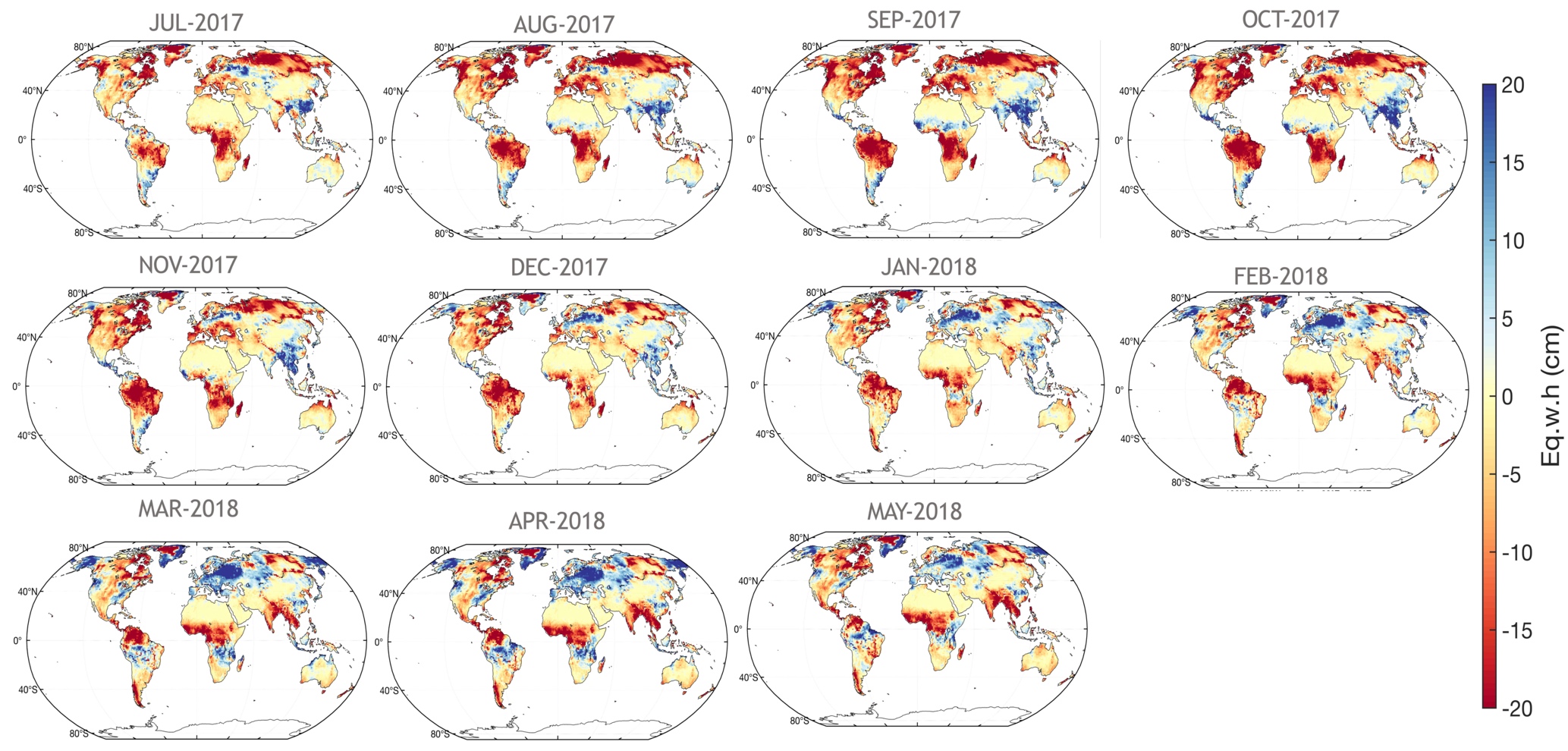


Figure S4. Total Water Storage (TWS) anomalies simulated by CLSM-F2.5 model during GRACE and GRACE-FO gap







Figure S5. The variability of the predicted signal and the residuals in cm unit. The predicted values are the fit of long-term variability, annual and semiannual with 2000 model for each component.


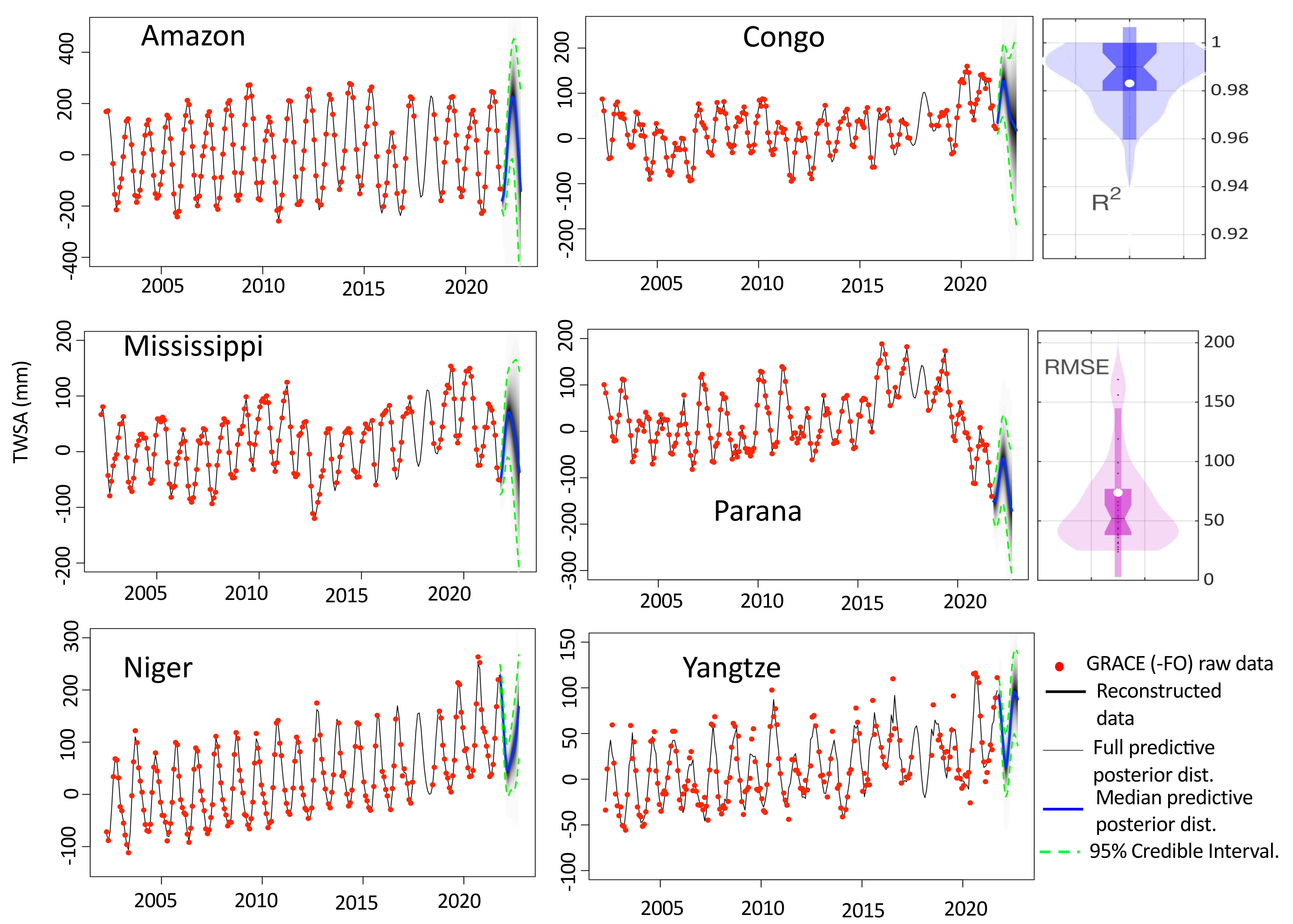


[a]

[d]

[c]

[b]

[g]

[f]

[e]

[h]

Figure S6. An example of the reconstructed GRACE like data represented by four examples (Amazon, Congo, Mississippi, Parana, Niger and Yangtze. The reconstructed data are in black lines (04/2002 to 09/2021), original time series are in red circles from CSR.M. Predictive posterior distributions of (4000) samples are in grey lines for the period (09/2021- 09/2022), with their median in blue lines, and 95% credible interval (dashed green lines) (a-f). The performance of the K-fold cross validations is summarized by r-square and RMSE tests (g-h) for 30 basins.

Figure S7.
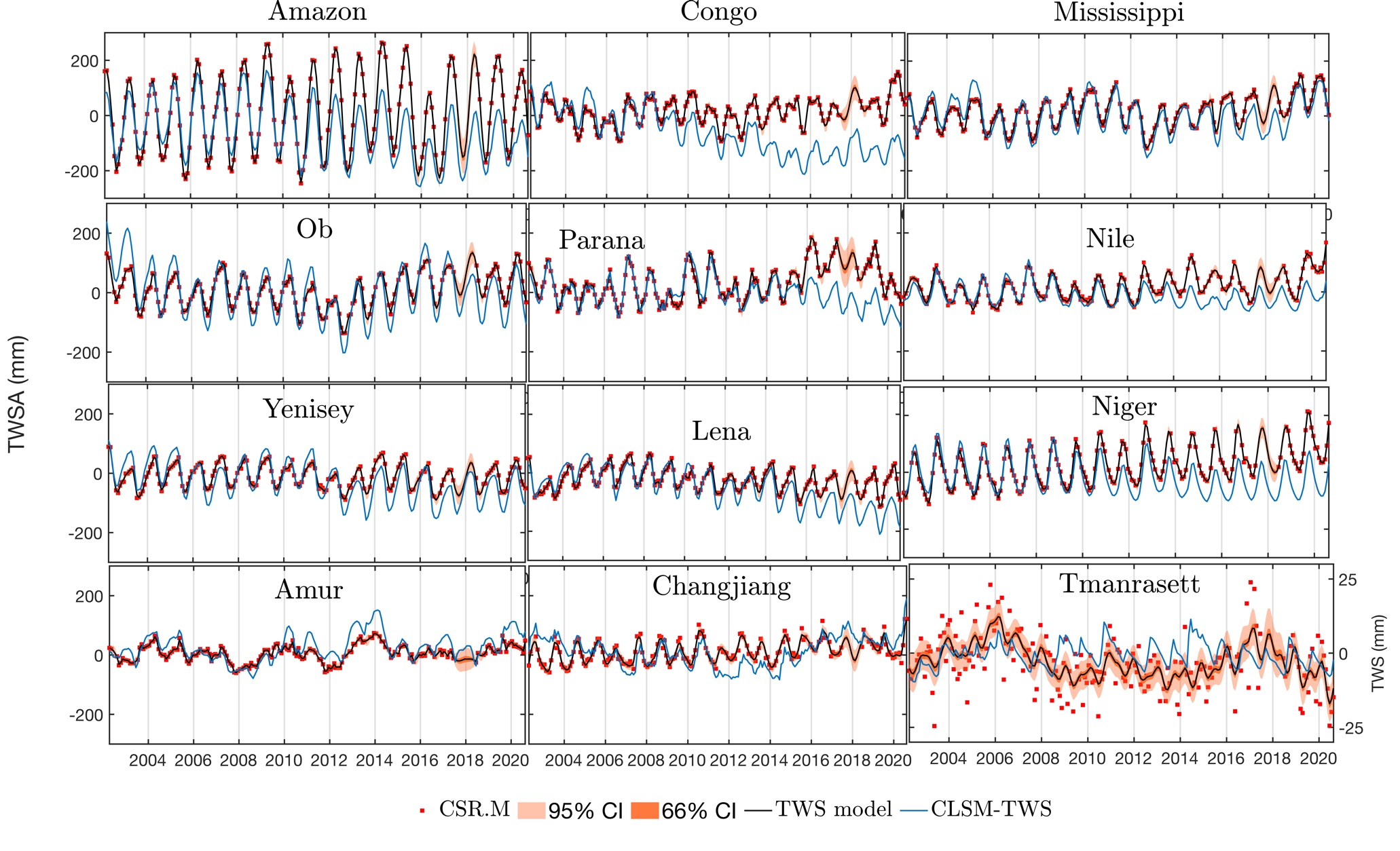
 Examples of filling the missing observation within and between GRACE missions for 12 hydrological basins. Red points are the original observations as calculated by the CSR mascon solutions; the black line is the reconstructed signal obtained after joining the median of the posterior of the temporal components (Figure S1), with two-level of credibility (66%, 95%), and the blue line is CLSM-TWS.

Table S1.  Model diagnostic tests for the reconstructed data using Coefficient of determinations (r^2^) for 30 hydrological basins. The comparison with the CLSM-TWS was measured for the whole period between 04/2002 and 08/2020 using four tests; NSE is the (Nash Sutcliff Efficiency), RMSE (Root Mean Square Errors), Std (Standard Deviations), and CC (Correlation Coefficient).

| ID | Name | Final TWS model | 5-Fold Cross Validation | | | GRACE (-FO) vs CLSM -TWS | | | |
| --- | --- | --- | --- | --- | --- | --- | --- | --- | --- |
|  |  | **(R^2^)** | **RMSE**  (km^3^) | **MAE**  (km^3^) | **R^2^** | **NSE** | **RMSE**  **(** km^3^**)** | **Std.ratio** | **CC** |
| 1 | Amazon | 1.00 | 392 | 391.6 | 0.7 | 0.64 | 344.47 | 1.49 | 0.96 |
| 2 | Congo | 0.99 | 156 | 156.1 | 0.4 | 0.67 | 103.94 | 0.96 | 0.83 |
| 3 | Mississippi | 1.00 | 99 | 99.0 | 0.6 | 0.87 | 73.12 | 0.84 | 0.94 |
| 4 | Ob | 0.99 | 77 | 76.9 | 0.7 | 0.79 | 121.24 | 0.65 | 0.93 |
| 5 | Parana | 0.99 | 169 | 168.5 | 0.4 | 0.39 | 108.79 | 1.17 | 0.76 |
| 6 | Nile | 0.98 | 119 | 118.7 | 0.4 | 0.63 | 59.47 | 1.23 | 0.87 |
| 7 | Yenisey | 0.97 | 44 | 43.6 | 0.7 | 0.77 | 65.26 | 0.74 | 0.89 |
| 8 | Lena | 0.98 | 54 | 54.2 | 0.7 | 0.77 | 51.94 | 0.82 | 0.88 |
| 9 | Niger | 0.99 | 59 | 59.2 | 0.8 | 0.92 | 34.66 | 1.04 | 0.96 |
| 10 | Amur | 0.97 | 66 | 65.6 | 0.1 | 0.59 | 57.67 | 0.56 | 0.81 |
| 11 | Changjiang | 0.88 | 63 | 62.7 | 0.4 | 0.18 | 85.16 | 0.61 | 0.46 |
| 12 | **Tmanrasett** | **0.41** | **16** | **16.3** | **0.1** | **-1.18** | **10.61** | **1.30** | **0.19** |
| 13 | Mackenzie | 0.99 | 52 | 51.9 | 0.6 | 0.67 | 43.28 | 0.91 | 0.82 |
| 14 | Volga | 0.99 | 38 | 37.9 | 0.8 | 0.66 | 107.10 | 0.49 | 0.92 |
| 15 | Zambezi | 1.00 | 77 | 76.8 | 0.7 | 0.64 | 78.57 | 1.30 | 0.90 |
| 16 | Lake Eyre | 0.95 | 36 | 36.5 | 0.1 | 0.73 | 18.77 | 1.06 | 0.88 |
| 17 | Nelson | 1.00 | 32 | 31.7 | 0.6 | 0.69 | 24.16 | 0.97 | 0.84 |
| 18 | St-Lawrence | 1.00 | 44 | 44.5 | 0.4 | 0.45 | 49.55 | 0.94 | 0.71 |
| 19 | Murray | 0.97 | 44 | 44.2 | 0.2 | 0.69 | 24.30 | 1.01 | 0.85 |
| 20 | Ganges | 1.00 | 77 | 76.9 | 0.5 | 0.71 | 46.05 | 1.26 | 0.91 |
| 21 | Orange | 0.97 | 24 | 24.1 | 0.2 | 0.71 | 14.10 | 0.79 | 0.84 |
| 22 | Indus | 0.99 | 28 | 27.7 | 0.4 | 0.23 | 21.84 | 1.44 | 0.80 |
| 23 | Chari | 1.00 | 49 | 49.4 | 0.7 | 0.86 | 25.70 | 1.09 | 0.94 |
| 24 | Orinoco | 1.00 | 90 | 89.8 | 0.7 | 0.78 | 52.77 | 1.28 | 0.94 |
| 25 | Tocantins | 0.99 | 77 | 77.3 | 0.7 | 0.81 | 42.87 | 1.31 | 0.97 |
| 26 | Yukon | 0.97 | 26 | 25.8 | 0.6 | 0.74 | 33.56 | 0.62 | 0.91 |
| 27 | Danube | 0.98 | 43 | 42.5 | 0.4 | 0.85 | 28.53 | 0.68 | 0.96 |
| 28 | Mekong | 0.99 | 36 | 36.2 | 0.8 | 0.90 | 36.61 | 0.92 | 0.95 |
| 29 | Cubango | 0.99 | 31 | 31.5 | 0.4 | 0.56 | 27.36 | 1.05 | 0.79 |
| 30 | Victoria Wiso | 0.99 | 39 | 38.6 | 0.1 | 0.74 | 19.39 | 1.07 | 0.88 |





Figure S8. Performance of the four reconstructed data for the global basins (186 basins , figure S9),  relative to this study and the CLSM model (de-trended data). The results for [Li et al. [2020]](#_ENREF_1) are available for the 37 basins only*.*


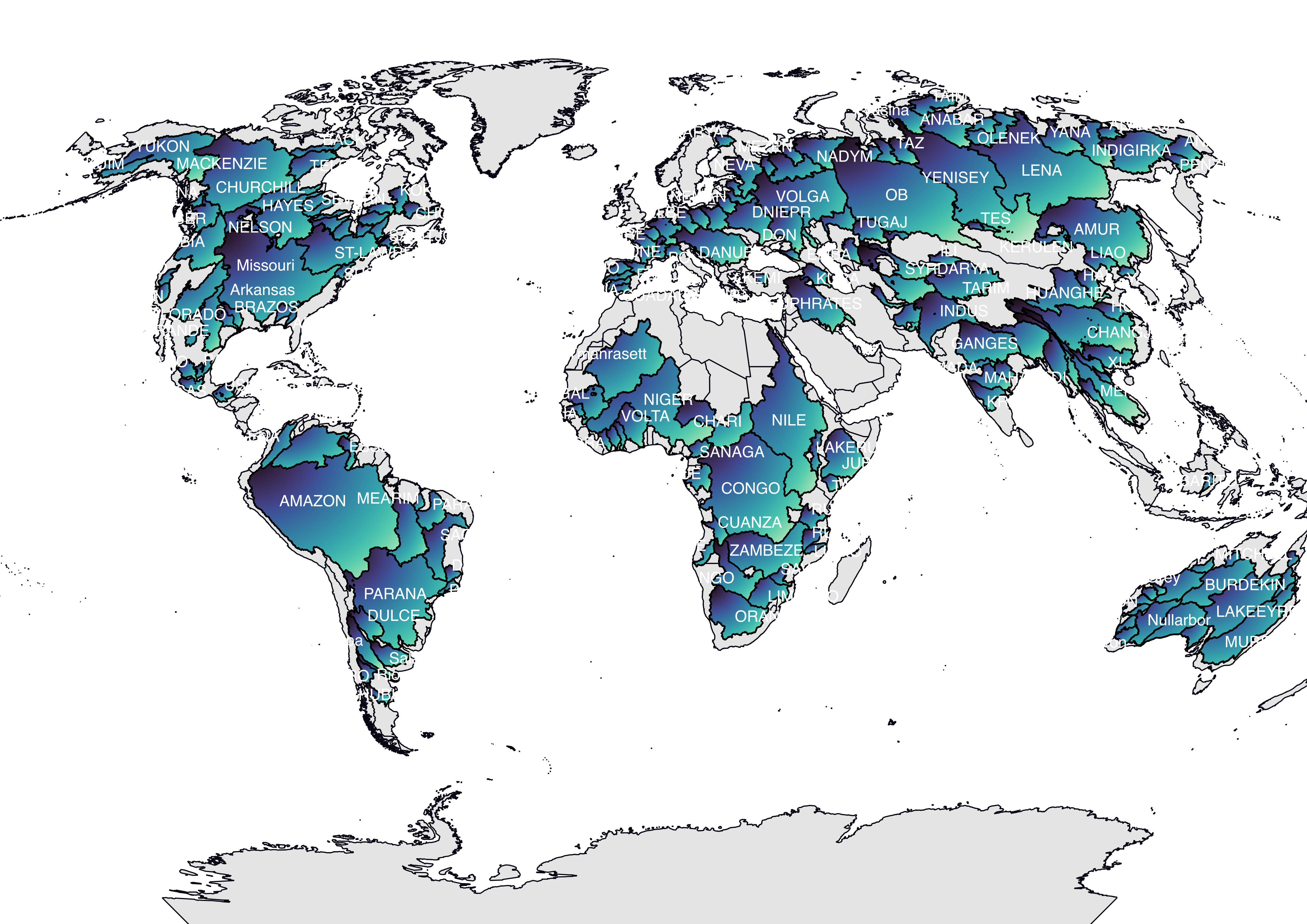


Figure S9. Location of 186 hydrological basins outlined used to compare the reconstructions data from four studies (Figure S8)

**References**

Li, F., J. Kusche, R. Rietbroek, Z. Wang, E. Forootan, K. Schulze, and C. Lück (2020), Comparison of data‐driven techniques to reconstruct (1992–2002) and predict (2017–2018) GRACE‐like gridded total water storage changes using climate inputs, *Water Resour Res*, *56*(5), e2019WR026551.
